# Patterns of adverse drug reaction signals in NAFDAC pharmacovigilance activities from January to June 2015: safety of drug use in Nigeria

**DOI:** 10.1002/prp2.427

**Published:** 2018-10-08

**Authors:** Olufunsho Awodele, Rebecca Aliu, Ibrahim Ali, Yetunde Oni, Christianah Mojisola Adeyeye

**Affiliations:** ^1^ Department of Pharmacology, Therapeutics & Toxicology College of Medicine University of Lagos Lagos Nigeria; ^2^ National Agency for Food and Drug Administration and Control (NAFDAC) Abuja Nigeria

**Keywords:** adverse drug event, adverse drug reaction, ARVs, NAFDAC, Nigeria, suspect drug

## Abstract

Adverse drug reactions (ADRs) are expected to be associated with an economic drain on the healthcare systems. The study was carried out to determine the occurrence of ADRs reported to NAFDAC Pharmacovigilance from January to June 2015, to illustrate the pattern of organ system affected by ADRs, to assess the completeness of ADR report, to determine the relationship between the occurrence of ADRs with suspect drugs and the use of concomitant drugs as well as to generate possible signals from the reported ADRs. A total number of 921 ADR cases reported from January to June 2015 were analyzed using SPSS version 22. A higher percentage of ADR reports were seen in females (65.5%). The highest percentages of reports (45.6%) were from the age range of 21‐40 years, most of the suspected drugs reported had both NAFDAC (50.2%) and batch number identification (65.6%). HIV (56.9%) was the most prevalent indication reported for using the suspected drug; Zidovudine/Lamivudine/Nevirapine combination (16.9%) was reported as the suspected drug with the highest occurrences of ADRs and generalized body itching (6.9%) as the most prevalent ADR. “General disorders” (47.3%) was the most predominant organ system affected by ADRs and Pharmacists were revealed as the highest reporters of ADRs (80.2%). Overall, patients on ARVs should be vigilantly followed up as they are mostly prone to ADRs. Adverse drug reaction reporting systems need to be robust and complete in order to be able to detect new drug alerts, possible signals and improve pharmacovigilance

**Abbreviations**
ACT: artemisinin‐based combination therapy
ADE: adverse drug event
ADRs: adverse drug reactions
ART: Antiretroviral therapy
ARV: Antiretroviral
CHEW: Community Health Extension Workers
FDA: Food and Drug Administration
ICSR: individual case study report
MHRA: Medicines and Healthcare products Regulatory Agency
NAFDAC: National Agency for Food Drug Administration and Control
NPC: National Pharmacovigilance Centre
SJS: Stephen Johnson Syndrome
UMC: Uppsala Monitoring Centre
WHO‐ART: World Health Organization Adverse Reaction Terminology
WHO: World Health Organization
ZPC: zonal pharmacovigilance centers

## 1 INTRODUCTION

The repeated occurrence of unexpected, serious adverse drug reactions (ADRs) over the years has attracted wide professional and public attention. This has cast doubt on the effectiveness and quality of drug safety surveillance systems.^1^ Adverse drug reactions (ADRs) represent an important risk for patients as they could cause significant disability and mortality, and are expected to be associated with an economic drain on the healthcare systems.^2^ Adverse drug reaction signals are reported information on possible causal relationships between an adverse event and a drug.^3^ A group of scientists proposed that the assessment of ADRs, therefore, is likely to be the most important aspect of drug treatment.^4^ ADRs are, in fact, responsible for around 4.9% of hospital admissions worldwide, and, in some cases, this number can be as high as 41.3%.^5^ There is thus no doubt that drug safety is an important public health problem.

Spontaneous reporting of suspected adverse drug reactions has long been the cornerstone of pharmacovigilance for the identification of early signals of problems of drug safety related to the use of medicines worldwide.^6^ Health professionals have contributed significantly to successful pharmacovigilance through spontaneous reporting. This enormously significant contribution has encouraged ongoing ascertainment of the benefit‐risk ratio of some drugs^7^, ^8^, as well as contributed to signal detection of unsuspected and unusual ADRs previously undetected during the initial evaluation of a drug.^9^, ^10^ Pharmacovigilance is an important and integral part of clinical research.^11^ It continues to play a crucial role in meeting the challenges posed by the ever increasing range and potency of medicines as it is a well‐known fact that no drug is completely free from adverse effects.

In Nigeria, the National Pharmacovigilance Centre (NPC) is domicile in National Agency for Food and Drugs Administration and Control (NAFDAC) and has the data bank of all reported adverse drug reactions in Nigeria.^3^ There are a bunch of examples of drugs, which have been detached as well as outlawed from the Nigerian market owing to reported adverse effects of drugs.^12^ Spontaneous reporting of ADRs to the NPC in Nigeria has prompted the timely withdrawal of toxic paracetamol adulterated with diethylene glycol that claimed the lives of some infants and young children in 2008.^13^, ^14^ It has also led to the ban of dipyrone in 2005 due to the frequent injection abscess and unexplained deaths associated with its use.^15^, ^16^ Hence, continuous postmarketing surveillance and signal detection from NAFDAC Pharmacovigilance database is important to guaranty the safety of patients.

The review of adverse drug reactions reported to NAFDAC in order to determine the patterns of adverse drug reaction signals in NAFDAC pharmacovigilance activities as well as explore information about new and unexpected adverse drug reactions reported is essential in safety of medicine assessment. This study is therefore aimed at determining the occurrence of ADRs reported to NAFDAC Pharmacovigilance, illustrating the pattern of organ system affected by ADRs reported, assessing the completeness of ADR reported data in NAFDAC Pharmacovigilance, determining the relationship between the occurrences of ADRs with suspect drugs as well as generate possible signals from the reported ADRs.

The outcome of this study will add to the pool of information available as regards ADRs and signals in NAFDAC and Uppsala Monitoring Centre (UMC). It will also form the epidemiological basis for certain regulatory decisions as affects the use of drugs.

## 2 METHODOLOGY

**Figure 1:**
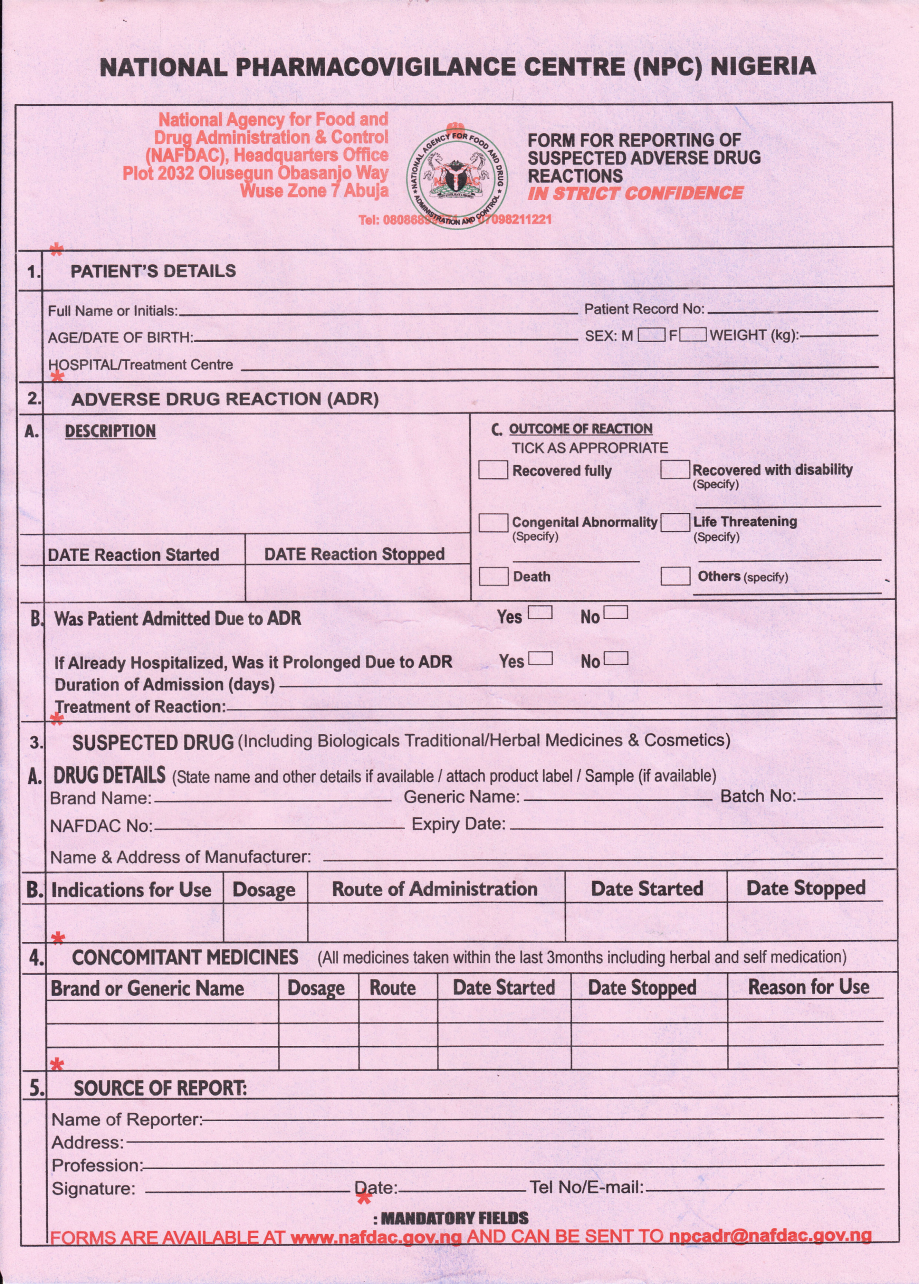
Adverse drug reaction reporting form

Spontaneous reporting of ADRs is practiced in Nigeria using a standard structured yellow form **(**Figure 1 **)** as recommended by the World Health Organization‐Uppsala Monitoring Centre (WHO‐UMC) in Sweden. The five general components of the form are patient's details, adverse drug reaction details, suspected drug details, concomitant medicines details, and sources of report. Healthcare providers and patients can send ADR reports to either the NPC, zonal pharmacovigilance centers (ZPCs), or NAFDAC state offices nationwide. All completed adverse drug reaction forms are submitted to NPC for documentation and analysis is done by experts. A filled yellow/adverse reaction form is known as the individual case study report (ICSR). The ADRs are coded on the basis of the WHO Adverse Reaction Terminology (WHO‐ART).^17^ The reports concluded to be ADRs are sent to UMC excluding the names of the patient and names of reporters for entry into the WHO Global Individual Case Safety Report database, VigiBase^®^.

### 2.1 Data abstraction

The ICSR of patients who experienced adverse drug reaction(s) from January 2015 to June 2015 were sourced from the NPC in Nigeria (NAFDAC) and data mining was done to obtain the following information: Demographic distribution of patients, batch and NAFDAC number identification for suspect drugs with ADRs, suspect drugs with ADRs (dosage form, specific indication for use, specific name, specific manufacturer), and ADR (specific type, duration, system organ classification, and outcome), reporter of ADR (institution and profession).

### 2.2 ADR outcome rating

Outcome of the ADR refers to the extent of resolution of the signs and symptoms of ADR as at the time the report was submitted to NPC. The outcomes were categorized as resolved, ongoing, resolving, life‐threatening, resolved with disability, and death.

### 2.3 Ethical considerations

The National Agency for Food and Drug Administration and Control (NAFDAC) approved the study.

### 2.4 Analysis

The data were analyzed with IBM SPSS statistics software, version 22. Descriptive statistics was used to summarize demographic distribution of patients, batch and NAFDAC number identification for suspect drugs with ADRs, suspect drugs with ADRs (dosage form, specific indication for use, specific name, specific manufacturer, country of manufacture), and ADR (specific type, duration, system organ classification, and outcome), reporter of ADR (institution and profession). Chi‐square test was used to test the statistical significance of categorical variables.

## 3 RESULTS

### 3.1 Demographic distribution of patients

A total number of 921 ADR cases were reported from January to June 2015. The demographic distribution of patients, batch and NAFDAC number identification for suspected drugs with ADRs (Table 1 **)** show a higher number of ADR reports in females (65.5%). The highest percentages of reports were from the age range of 21‐40 years (45.6%).

**Table 1:** Demographic distribution of patients, batch, and NAFDAC number identification for suspected drugs with ADRs

Variable	Frequency	Percentage
Gender		
Male	318	34.5
Female	603	65.5
Age (years)		
1‐20	92	10.0
21‐40	420	45.6
41‐60	170	18.5
61‐80	20	2.2
81‐100	15	1.6
Adult (unspecified age)	204	22.1
Total	921	100.0
NAFDAC number of suspected drug reported		
Yes	469	50.2
No	466	49.8
Total	935	100.0
Batch number of suspected drug reported		
Yes	613	65.6
No	322	34.4
Total	935	100.0

The percentage of suspected drugs reported to have NAFDAC numbers (50.2%) were similar to the percentage without NAFDAC numbers (49.8%). However, a higher percentage of reported drugs were with batch number identification (65.6%).

### 3.2 Specific indication for using the suspected drug(s)

The profile of specific indication for using the suspected drugs (Table 2) reveal that HIV (56.9%) was the most prevalent indication reported for using the suspected drug, followed by fever/malaria (6.9%), tuberculosis (5.7%), prevention of one ailment or the other (3.1%), etc. ‘Others’ represent a classification of indications only reported once.

**Table 2:** Profile of specific indication for using the suspected drug(s)

Indication for use reported	Frequency	Percentage
Yes	875	93.8
No	58	6.2
Total	933	100.0
Specific indication		
HIV	531	56.9
Fever/Malaria	64	6.9
Tuberculosis	53	5.7
Prophylaxis	29	3.1
Body pain	26	2.8
Bacterial infection/skin infection	18	1.9
Hypertension	14	1.5
Hepatitis	9	0.9
Cough	9	0.9
Cough/Cold/Catarrh	8	0.8
Headache	8	0.8
Cancer	8	0.8
Abdominal pain	8	0.8
Waist pain	4	0.4
Diabetes mellitus	4	0.4
Rheumatism	3	0.3
Urinary tract infection	3	0.3
Diarrhea	3	0.3
Typhoid/salmonella	3	0.3
Catarrh	3	0.3
Peptic ulcer	3	0.3
Pelvic inflammatory disease	2	0.2
Helminthiasis	2	0.2
Anemia	2	0.2
Psychosis	2	0.2
Infertility	2	0.2
Osteoarthritis	2	0.2
“Others”	110	11.7
Total	933	100

### 3.3 Suspected drugs causing ADRs

The profile of suspected drugs with ADRs (Table 3) showed that zidovudine/lamivudine/nevirapine (16.9%) combination was reported 150 times causing the highest episode of ADRs, followed by efavirenz reported seventy‐eight (78) times (8.8%). ‘Others’ category was reserved for drugs with frequencies less than four.

**Table 3:** Profile of suspected drugs causing ADRs

Suspected drug(s) reported	Frequency	Percent
Yes	890	95.3
No	44	4.7
Total	934	100.0
Specific suspected drug(s)		
Zidovudine/Lamivudine/Nevirapine	150	16.9
Efavirenz	78	8.8
Nevirapine	76	8.5
Zidovudine	58	6.5
Tenofovir/Efavirenz/Lamivudine	54	6.1
Artesunate/septrin	42	5.4
Zidovudine/Lamivudine	28	3.1
Artemeter‐lumefantrine	21	2.4
Tenofovir/Lamivudine	19	2.1
Tramadol	19	2.1
Levofloxacin	18	2.0
Sulphadoxine/Pyrimethamine	14	1.6
Ciprofloxacin	11	1.2
Prothionamide	9	1.0
Diclofenac	8	0.9
Kanamycin	8	0.9
Tenofovir/Lamivudine/Nevirapine	8	0.9
Cycloserin	7	0.8
Interferon Alpha	7	0.8
Tenofovir alone	7	0.8
Insulin	7	0.8
Zidovudine/Efavirenz/Lamivudine	6	0.7
5% Dextrose saline	6	0.7
Tenofovir/Emtricitabine	6	0.7
Paracetamol	5	0.6
Metronidazole	5	0.6
Zidovudine/Nevirapine	5	0.6
Chloroquine	5	0.6
Dihydroartemisinine/Piperazine	4	0.4
Ibuprofen	4	0.4
Sodium chloride	4	0.4
Ceftriazone	4	0.4
Cefuroxime	4	0.4
Kanamycin/cycloserin/prothionamide	4	0.4
Erythromycin	4	0.4
Prochlorperazine	4	0.4
‘Others’	171	19.2
Total	890	100

### 3.4 Reported ADRs with suspected drugs

It was observed from the study (Table 4) that the most prevalent ADR was “generalized body itching” being reported 65 times (6.9%), “rash all over the body” was reported 49 times (5.3%), and “anemia” was reported 35 times (3.8%). “Others” category was reserved for ADRs with frequencies less than four.

**Table 4:** Profile of reported ADRs with suspected drug

ADRs reported	Frequency	Percentage
Yes	931	99.6
No	4	0.4
Total	935	100.0
If yes, specific ADRs		
Generalized body itching	65	6.9
Rash all over the body	49	5.3
Anemia	35	3.8
Vomiting	34	3.7
Dizziness	31	3.3
Headache	22	2.4
Stomach pain/abdominal discomfort	18	1.9
Rash/pruritus	15	1.6
Muscle pains	12	1.3
Steven Johnson syndrome	12	1.3
Peripheral neuropathy	10	1.1
Rigor	9	1.0
Dyspepsia	7	0.8
Weakness/dizziness	6	0.6
Increased appetite	6	0.6
Itching and skin eruption	6	0.6
Fatique/weakness	5	0.5
Hyperpigmentation	5	0.5
Hearing loss	4	0.4
Lipodystrophy	4	0.4
Swollen eye	4	0.4
Purging/diarrhea	4	0.4
Insomnia	4	0.4
Dizziness/headache/blurred vision/body weakness/fatigue	4	0.4
Nightmare	4	0.4
Paresthesia/numbness	4	0.4
“Others”	552	59.3
Total	931	100

### 3.5 Organ system classification of reported ADRs

Table 5 shows a detailed list of system organ classification for the reported ADRs. Findings from the study revealed that, “general disorders” was the most predominant organ system affected by ADRs, being reported 431 times (47.3%). “Skin and subcutaneous skin disorders” was reported 238 times (26.1%). “Vascular disorders” was least reported being reported only twice (0.2%).

**Table 5:** System organ classification of reported ADRs

System classification	Frequency	Percentage
General disorders	431	47.3
Skin and subcutaneous skin disorders	238	26.1
Gastrointestinal disorders	74	8.1
Blood and lymphatic system disorders	50	5.5
Eye disorders	21	2.3
Respiratory disorders	12	1.3
Hepatobiliary disorders	9	1.0
Nervous system disorders	14	1.5
Reproductive system and breast disorders	8	0.9
Endocrine disorders	14	1.5
Musculoskeletal and connective disorders	15	1.6
Psychiatric disorders	4	0.4
Renal and injury disorders	8	0.9
Cardiac disorder	5	0.5
Metabolic and nutritional disorders	3	0.3
Ear and Labyrinths disorders	3	0.3
Vascular disorder	2	0.2
Total	911	100.0

### 3.6 Source of suspected drug and nature of outcome of ADRs reported

The findings of the study revealed that most of the suspected drugs reported were sourced from the Hospital Pharmacy (Table 6) being reported 746 times (86.4%). Community pharmacy was reported 99 times (11.5%). The open market was reported twice (0.3%) as source of suspected drug.

**Table 6:** Source of suspected drug and outcome of ADRs reported

	Frequency	Percentage
Source of suspected drug(s) with ADRs		
Hospital Pharmacy	746	86.4
Community Pharmacy	99	11.5
Company	16	1.9
Open market	2	0.2
Health office	1	0.1
Total	864	100.0
Outcome of ADRs reported		
Yes	509	54.1
No	426	45.6
Total	935	100.0
If yes, nature of outcome for reported ADRs		
Resolved	340	66.8
Ongoing	100	19.6
Resolving	26	5.1
Life‐threatening	25	4.9
Resolved with disability	10	2.0
Death	8	1.6
Total	509	100.0

### 3.7 Profession of reporter of ADRs

The findings in this study showed that Pharmacists (Table 7) reported ADRs the most. The “others” category refer to the pharmacy focal person, pharmacovigilance officer, human resource practitioner, media organization, house wife, self‐employed person, hematolgist, engineer, teacher, as well as medical social work officer which each reported once.

**Table 7:** Profile of profession of reporters of ADRs

	Frequency	Percentage
Profession reported		
Yes	705	92.8
No	55	7.2
Total	760	100.0
Specific profession of reporter of ADRs		
Pharmacist	672	80.2
Non health professional	34	4.1
Student	24	2.9
Pharm Technician	17	2.0
Physician/medical practitioner	15	1.8
Medical doctor	14	1.7
Civil servants	14	1.7
Other health professional	10	1.4
Pharmacologist	6	0.9
Nurse	5	3.8
Applicant/student	4	3.0
Business woman/trader	3	2.3
Data entering Clerk	3	0.4
CHEW	2	0.3
Others	15	1.8
Total	838	100.0

### 3.8 Dosage form of suspected drug with ADRs

Table 8 shows the frequency of ADRs associated with different routes of administration. The most prevalent dosage form which caused an ADR was the oral dosage form (Tablets).

**Table 8:** Profile of dosage forms for administration of suspected drugs

	Frequency	Percentage
Tablet	810	89.7
Intravenous	53	5.9
Syrup	14	1.6
Suspension	8	0.9
Capsule	7	0.8
Topical	3	0.3
Eye drop	2	0.2
Intramuscular	2	0.2
Subcutaneous	1	0.1
Inhalation	1	0.1
Transdermal	1	0.1
Transplacental	1	0.1
Total	903	100.0

### 3.9 Relationship of relevant variables and occurrence of ADR with suspected drugs

Table 9 shows a profile of the relationship between variables (age, gender, batch and NAFDAC number specification, concomitant drug use) and the occurrence of ADR in the first quarter while table shows the result for relationship between variables in the second quarter. There were no statistically significant association (*P* ≥ 0.05) between age, gender, batch number of suspected drugs, NAFDAC number on suspected drugs, concomitant drugs with suspected drugs, and occurrence of ADRs with suspected drugs.

**Table 9:** (A) Relationship of relevant variables and occurrence of ADR with suspected drugs (First quarter). (B) Relationship of relevant variables and occurrence of ADRs with suspected drugs (Second quarter)

Variables	Occurrence/experience of ADRs with suspected drug(s), N (%)		χ^2^	*P*‐Value
	Yes	No		
(A)				
Age (year)			4.012	0.548
1‐20	65 (100.0)	0 (0.0)		
21‐40	362 (8.9)	0 (0.0)		
41‐60	143 (100.0)	0 (0.0)		
61‐80	12 (100.0)	0 (0.0)		
81‐100	3 (100.0)	0 (0.0)		
Adult unspecified	145 (100.0)	1 (0.0)		
Gender			0.983	0.321
Male	248 (100.0)	0 (0.0%		
Female	504 (99.6)	2 (0.4)		
Batch number for suspected drug			4.592	0.101
Yes	529 (100.0)	0 (0.0)		
No	229 (99.1)	2 (0.9)		
NAFDAC number for suspected drug			2.337	0.311
Yes	409 (100.0)	0 (0.0)		
No	349 (99.4)	2 (0.6)		
Concomitant drug(s) used with suspected drug			4.586	0.101
Yes	526 (100.0)	0 (0.0)		
No	228 (99.1)	2 (0.9)		
Outcome of ADRs reported			3.223	0.666
Resolved	250 (100.0)	0 (0.0)		
Ongoing	92 (98.9)	1 (1.1)		
Resolved with disability	9 (100.0)	0 (0.0)		
Resolving	22 (100.0)	0 (0.0)		
Life‐threatening	16 (100.0)	0 (0.0)		
Death	2 (100.0)	0 (0.0)		

Variables	Occurrence/experienced of ADRs with suspected drug(s), N (%)		Chi‐square (χ^2^)	*P*‐Value
	Yes	No		
(B)				
Batch number for the suspected drug			0.950	0.330
Yes	85 (100.0)	0 (0.0)		
No	89 (98.9)	1 (1.1)		
NAFDAC number for the suspected drug			0.538	0.463
Yes	61 (100.0)	0 (0.0)		
No	113 (99.1)	1 (0.9)		
Concomitant drug(s) used with suspected drug			1.194	0.274
Yes	95 (100.0)	0 (0.0)		
No	79 (98.8)	1 (1.2)		

Level of significance *P* < 0.05

### 3.10 The pattern and profile of reported adverse drug reactions of Zidovudine/Lamivudine/Nevirapine combination

A wide range of ADRs were reported for Zidovudine/Lamivudine/Nevirapine. Discoloration of finger nails, nausea, and vomiting were reported more than once as ADRs when Zidovudine/Lamivudine/Nevirapine was administered without a concomitant drug. Skin rash was the most reported ADR with Zidovudine/Lamivudine/Nevirapine use being reported 25 times (16.9%), followed by anemia/fatigue which was reported 23 times (15.54%) and headache reported 16 times (10.81%). From the report, headaches and increased appetite are the most commonly reported ADRs when cotrimoxazole is coadministered with Zidovudine/Lamivudine/Nevirapine. Urination of blood, swelling of face, dizziness; swollen legs, inability to walk, cough; are the life‐threatening ADRs reported with Zidovudine/Lamivudine/Nevirapine combination alone. Life‐threatening ADRs with concomitant drugs include severe anemia (most common), Stephen Johnson Syndrome (SJS), generalized body itching, and cough.

It was inferred from the report that 19.59% (29) reported ADRs resolved without any sequelae, 4.73% (7) were life‐threatening. Zidovudine/lamivudine/nevirapine with cotrimoxazole alone (concomitant drug) resulted in 40.54% (17) of ADRs report.

### 3.11 The pattern and profile of reported adverse drug reactions of ACT

Table 10 shows the result of the adverse drug reactions reported for artemether/lumefantrine, artemether/piperaquine, artesunate/amodiaquine, dihydroartemisinin/piperaquine, and dihydroartemisinin/piperazine, as well as the concomitant drug(s) used with this drug combinations and the outcomes of the adverse drug reactions.

**Table 10:** Adverse drug reactions reported with artemesinin‐based combination therapy (ACT)

Suspected drug	Adverse reactions	Concomitant drug(s)	Outcome of ADR
Artemether/Lumefantrine	Vomiting; weakness, dizziness; cough; dizziness, fainting; itching	None	Resolved
	Body weakness, dizziness, lack of appetite	None	Resolving
	Generalized itching; black patches on skin; reddish rash; Palpitation; treatment failure (2); appearance of boils on the face	None	Not documented
	Severe itching, swelling around the ears and head	Chlorpheniramine/hydrocortisone	Resolved
	Swelling of face and lips	Lisinopril/nifedipine/moduretic	Resolved
	Dizziness, weakness, dim vision, almost collapsing	Paracetamol	Resolved
	Fever, vomiting	Albendazole/fesolate	Resolved
	Pruritus	Diclofenac/vitamin c/piroxicam, misoprostol/fesolate/vitamin b complex/zidovulam/lamivudine/nevirapine	Not documented
	Pyrexia, dizziness; dizziness, malaise	Ergotamine/metformin/glimepiride	Not documented
Artemether/piperaquine	Generalized papilla rash with itching	None	Resolved
	Papilla rash, reddish eye, itching, pink lips with blisters accompanied with stomach discomfort	None	Resolving
	Generalized papilla rash with itching	None	Not documented
	Severe itching and discomfort, generalized body rash	Paracetamol	Resolved
Artesunate/amodiaquine	Neck pain, serious headache, weakness of the body and back bone	None	Resolved
	Vomiting, hypoglycemia, very weak	Paracetamol	Resolved
Dihydroartemisinin/piperaquine	Severe abdominal pain, restlessness, difficulty in breathing, chest tightness	None	Resolved
Dihydroartemisinin/piperazine	Itching on the feet and palm	Paracetamol/supplements	Resolved

Dizziness is the most common specific ADR reported for artemether/lumefantrine, while treatment failure was reported twice. Papilla rash is the most reported specific ADR for artemether/piperaquine. No serious ADR was documented for all the ACTs reported that is all the ADRs reported resolved without sequelae.

## 4 DISCUSSION

The biological differences of males and females can affect the action of many drugs. The anatomical and physiological differences are body weight, body composition, gastrointestinal tract factors, liver metabolism, and renal function. Women in comparison to men have lower bodyweight and organ size, more body fat, different gastric motility and lower glomerular filtration rate. These differences can affect the way the body deals with drugs by altering the pharmacokinetics and pharmacodynamics of the drugs including drug absorption, distribution, metabolism and elimination.^18^ The findings revealed that females (65.5%) were reported to have more ADRs. This is in line with several other studies which have suggested that a female preponderance in the overall frequency of adverse drug reactions may be present, in that female patients have more ADRs.^19^, ^20^, ^21^ Gender may influence drug utilization and susceptibility to, presentation of, and detection of adverse drug reactions, although the results of this study showed that the influence is not statistically significant (*P* > 0.05). The lack of association may be due to a large proportion of reports from females that were neither pregnant nor breastfeeding as pregnancy is a known risk factor for ADRs occurrence.^18^, ^22^

Age has a significant effect on development of ADRs, especially the extreme ages that is pediatric and geriatric patients as these categories of patients are not usually studied extensively during clinical trials.^4^ The findings in this study are however not in tandem with the aforementioned. The study revealed age range of 21‐40 (45.6%) as the most prevalent reported age of patients. This may be as a result of underreporting of ADRs especially in children where ADRs could easily mimic other diseases. The results of this study are, however, consistent with recently published investigations conducted by Awodele et al^3^ which revealed age range of 31‐40 as the most prevalent reported age of patients with ADRs. Also in corroboration with these studies is the observation from previous studies of Agu et al^23^ and Agu and Oparah^24^ which reported 35.5 years as the mean age of patients reported to have adverse reactions to antiretroviral agents.

A higher percentage of drugs reported with ADRs had both NAFDAC (50.2%) and batch number (65.6%) clearly reported. This might go a long way to explain that, the presence of these numbers (NAFDAC and batch) which should ordinarily serve as a means of detecting authenticity, is not enough to guaranty safety of the drugs hence emphasizing the need for continuous drug monitoring.

HIV (56.9%) was the most reported specific indication for using the suspected drug. This is in positive correlation with the study of Awodele et al^3^ who also reported HIV (63.3%) as the highest indication for using the suspected drug. Generalized body itching (6.9%) was the most reported ADR, followed by rash all over the body (5.3%). These results corroborated previous research findings that skin rash and peripheral neuropathy were common ADRs in Antiretroviral therapy (ART) patients.^25^, ^26^ Eluwa et al^27^ reported ADR incidence rate of 4.6/100 person‐years; and commonest ADRs were pain (30%) and skin rash (18%). The study of Oreagba et al^28^ also documented skin reactions and rashes to be common ADRs with antiretroviral combination containing Zidovudine. These observations are consolidating the reports from this study which revealed that Zidovudine/lamivudine/nevirapine (16.9%) combination was the most reported suspected drug.

Findings from this study also revealed that no serious ADRs were reported for artemisinin‐based combination therapy (ACTs), that is there was no report of life‐threatening adverse drug reactions that could warrant termination of treatment or drug use although treatment failure was reported twice. This supports findings from China, Thailand, South East Asia and other African countries where ACTs have been used extensively and were found to be relatively safe and well tolerated.^29^, ^30^, ^31^, ^32^ This is also in line with the prospective study of Belhekar et al^33^ who suggested that ADRs from ACTs were of moderate intensity with the ADRs most commonly reported when chloroquine was prescribed as concomitant drug. However, this study reveals that there is no statistically significant association between the use of suspect drugs and concomitant drugs in ADRs occurrence. More surveillance in this regard is, however, advocated and quality of reports should also be ensured.

The tablet dosage form (61.3%) is the most reported dosage form of suspected drug causing ADR followed by intravenous dosage form (27.1%). This result is consistent with the known facts that tablets are the most recommended dosage forms and even most available for self‐medication. The intravenous dosage form on the other hand is the dosage form most prone to ADRs.

Pharmacists play a vital role in every step of the pharmacovigilance process^34^ and available data indicate that the introduction of nurses and pharmacists reporting is proving to be very useful (Morrison et al^35^ and van Grootheest et al^36^. This is evident by the findings in this study which revealed that Pharmacists (82.7%) were the highest reporters of ADRs to NAFDAC Pharmacovigilance. Justifying the aforementioned, Medicines and Healthcare products Regulatory Agency (MHRA) data revealed that the number of reports received from general practitioners (Doctors) in the last few years have been significantly low.^37^ There has not yet been any research into why this has occurred although speculation might pin point increased workload and administration or a presumption that others are reporting as possible reasons for this decline.

“General disorders” was the organ system (51.1%) reported to be most affected by ADRs, this is followed by skin and subcutaneous skin disorders (24.9%) and gastrointestinal disorders (7.6%). This result is consistent with documented studies carried out in Sweden, which states that ADRs were most frequently gastrointestinal (21.6%) or general disorders (12.3%).^38^

It is worth mentioning that most of the reports submitted to NAFDAC Pharmacovigilance were incomplete as they lacked necessary information like date the ADR started/stopped, suspected drug used along other requirements to validate the form. Of the 935 reported ADRs from January to June, only 509 had reported outcomes and 66.8% of the reported ADRs resolved. These findings are consistent with the previous studies reporting incompleteness of ADR forms submitted to pharmacovigilance centers in Mexico^39^ and Saudi Arabia,^40^ and those submitted to a pharmaceutical company in Italy.^41^ Incomplete ADR information may limit the effectiveness and full potential of analysis of reports. The NPC local database is used to store all reports received irrespective of their completeness status. Since the NPC has no rejection policy for incomplete suspected ADR reports, timely evaluation of the received suspected ADR reports should be considered as a means of early identification of incomplete reports. Reporters should be reached via repeated email, phone calls, or visits, and encouraged with incentives to providing missing details from the reports. Continuous pharmacovigilance education for healthcare professionals should emphasize the importance of completing the ADR report forms when reporting.^28^

## 5 CONCLUSION

The occurrence of ADRs reported in this study are comparable with those reported by other studies in Nigeria. Given the limitations of clinical trials in identifying rare and delayed ADRs, and the need for comprehensive drug safety profiles, the importance of reporting ADRs cannot be overemphasized and prompt recognition as well as reporting will go a long way in minimizing the occurrence of Adverse drug reactions. More surveillance is advocated to ascertain the consistency of the observed ADRs. Further training on appropriate reporting of ADRS is needed to ensure completeness of the reported ADRs thus establishing appropriate signals.

## DISCLOSURE

None declared.
